# Balancing efficiency, equity and feasibility of HIV treatment in South Africa – development of programmatic guidance

**DOI:** 10.1186/1478-7547-11-26

**Published:** 2013-10-09

**Authors:** Rob Baltussen, Evelinn Mikkelsen, Noor Tromp, AnneKarin Hurtig, Jens Byskov, Øystein Olsen, Kristine Bærøe, Jan A Hontelez, Jerome Singh, Ole F Norheim

**Affiliations:** 1 Department of Primary and Community Care, Radboud University Nijmegen Medical Centre, Nijmegen, The Netherlands; 2 Deparment of Public Health and Clinical Medicine Umeå University, Umeå International School of Public Health, Umeå, Sweden; 3 Centre for Health Research and Development, Faculty of Life Sciences, University of Copenhagen, Frederiksberg, Denmark; 4 Department of Global Public Health and Primary Care University of Bergen, Bergen, Norway; 5 Department of Public Health, Erasmus MC, University Medical Center Rotterdam, Rotterdam, The Netherlands; 6 Africa Centre for Health and Population Studies, University of KwaZulu-Natal, Mtubatuba, South Africa; 7 Centre for the AIDS Programme of Research in South Africa, Durban, South Africa; 8 Department of Public Health Sciences and Joint Centre for Bioethics, University of Toronto, Toronto, Canada; 9 Howard College School of Law, University of KwaZulu-Natal, Durban, South Africa

**Keywords:** Antiretroviral therapy, Technology assessment, Program evaluation, Cost-effectiveness, Ethics

## Abstract

South Africa, the country with the largest HIV epidemic worldwide, has been scaling up treatment since 2003 and is rapidly expanding its eligibility criteria. The HIV treatment programme has achieved significant results, and had 1.8 million people on treatment per 2011. Despite these achievements, it is now facing major concerns regarding (i) *efficiency*: alternative treatment policies may save more lives for the same budget; (ii) *equity*: there are large inequalities in who receives treatment; (iii) *feasibility*: still only 52% of the eligible population receives treatment.

Hence, decisions on the design of the present HIV treatment programme in South Africa can be considered suboptimal. We argue there are two fundamental reasons to this. First, while there is a rapidly growing evidence-base to guide priority setting decisions on HIV treatment, its included studies typically consider only one criterion at a time and thus fail to capture the broad range of values that stakeholders have. Second, priority setting on HIV treatment is a highly political process but it seems no adequate participatory processes are in place to incorporate stakeholders’ views and evidences of all sorts.

We propose an alternative approach that provides a better evidence base and outlines a fair policy process to improve priority setting in HIV treatment. The approach integrates two increasingly important frameworks on health care priority setting: accountability for reasonableness (A4R) to foster procedural fairness, and multi-criteria decision analysis (MCDA) to construct an evidence-base on the feasibility, efficiency, and equity of programme options including trade-offs. The approach provides programmatic guidance on the choice of treatment strategies at various decisions levels based on a sound conceptual framework, and holds large potential to improve HIV priority setting in South Africa.

## Introduction

With 5.7 million HIV-positive people, South Africa is the country with the largest HIV epidemic worldwide [1]. In recent years, the country has gradually expanded its eligibility criteria for treatment initiation in line with the World Health Organization (WHO) guidelines [2-6]. It is now rapidly scaling up its treatment programme aiming to cover all HIV-infected people with a CD4 cell count of ≤350 μl patients with a TB co-infection, and HIV-infected pregnant women irrespective of CD4 cell count [4,7]. The treatment programme has achieved significant results: it is the largest programme of its kind in the world, with approximately 1.8 million people on HIV treatment [8].

Despite these achievements, the present HIV programme is not optimal in three important areas. First, there are concerns about whether the current treatment strategy is most *efficient.* Research suggests that alternative policies such as universal testing and immediate treatment of all HIV-infected patients (UTT) [9] and targeting specific risk groups [10,11], would be more efficient than the present programme. Second, concerns exist regarding the *equity* of the distribution of ART across population groups – recent reviews show that e.g. men and children have less access to treatment than women [8,12]. Third, there are concerns about the programme *feasibility* given the severely limited capacity of the health system. In 2011, 3.4 million people were eligible for treatment in South Africa, and despite the achievements in scaling up the treatment programme, yet only 52% of them received it [8]. This “treatment gap” is related to funding constraints, but also due to staff shortages [13], and it will increase with more people surviving on treatment [13,14]. National health authorities acknowledge these concerns [4] but have not yet developed and implemented treatment guidelines that address these. This results in ad-hoc priority setting practices (where some clinicians treat patients on a first-come first-serve basis while others give preference to the most severely ill) and waiting lists in parts of the country [15].

In this paper, we argue that the above observations are related to suboptimal decisions on the design of the present HIV treatment programme. We argue there are two fundamental reasons to this. First, while there is a rapidly growing evidence-base to guide priority setting decisions on HIV treatment, its included studies typically consider only one criterion at a time and thus fail to capture the broad range of values that stakeholders have. Second, priority setting on HIV treatment is a highly political process but it seems no adequate participatory processes are in place to incorporate stakeholders’ views and evidences of all sorts.

In the paper, we first outline the scientific evidence-base on HIV treatment priority setting in South Africa, in terms of efficiency, equity and feasibility. We continue by proposing an alternative approach based on the combination of two innovative and increasingly important frameworks for health care priority setting: *accountability for reasonableness (A4R)* to foster fair priority setting processes, and *multi-criteria decision analysis (MCDA)* to foster rational priority setting.

The WHO has recently also recognised the need to trade-off the mentioned efficiency, equity and feasibility concerns [16], resulting in “Programmatic guidelines on HIV treatment” issued in July 2013 [17]. This paper contributes to these guidelines by providing a practical lead way for making these difficult priority setting decisions at various decision levels, based on a firm conceptual framework.

### Present approaches to HIV treatment priority setting

Present studies on HIV treatment in South Africa priority setting typically focus on one of the following criteria.

### Efficiency

Lately, a wide range of mathematical modelling studies have analysed the population health effects as well as costs and cost-effectiveness (or efficiency) of early versus late onset of treatment, many incorporating the transmission benefits of ART [11,18-30]. Although models agree that HIV incidence can be substantially reduced through expanded access to ART, models differ substantially on predicted impact and cost-effectiveness of such an intervention [9,31]. In addition, there is a growing interest in tailoring HIV treatment guidelines to most efficiently target programmes [10,11].

### Equity

Generally speaking, equity in health care pertains to judgements about distributive equality and the notion that every individual should have a “fair chance to live a full healthy life” [32]. Yet, with severely constrained resources as in HIV treatment in South Africa, difficult ethical choices need to be made on whom is prioritized for treatment. Only a few studies give normative guidance on this subject. More specifically, Cleary *et al.* use the concept of “communitarian claims” in which an individual is viewed as having a claim on health care due to being a member of a community or society—and by extension, society has some obligation to provide the care [33]. Claim strength is said to be affected by the severity of disease (sicker patients would be prioritized for moral reasons) or the individual capacity to benefit (patients with a better prognosis would be prioritized as this would lead to better clinical outcomes). Another claim stems from the impact of the programme on population health (patients would be prioritized whose treatment contributes most to reduction of the epidemic). Obviously, these above factors lead to conflicting recommendations on treatment initiation, particularly regarding whether this should be early or late in the course of disease. Other factors influencing claims include the “social context” of those in need. Kimmel *et al.*, [15] showed that professionals in South Africa support prioritizing individual patients based on treatment adherence, pregnancy status, and severity of illness.

Scholars take different positions when it comes to claim strength in the use of antiretrovirals for treatment or for prevention. Brock and Wikler argue that “the strongest moral imperative directs us to giving priority to saving the most lives (..) even if this means lowering the priority given to the goal of universal access to treatment, to provide maximum protection from HIV infection” [34]. In response, Macklin and Cowan reason that “it is unethical to deliberately watch patients with treatable HIV/AIDS worsen and die (..) if medication for treatment are diverted to preexposure prophylaxis” [35]. Alternatively, Singh proposes that a state’s “minimum core obligation” be used as a guiding principle in HIV programmes. This would protect the interest of all people, and as a consequence, antiretrovirals should not be exclusively used for treatment but also for prevention of HIV among, e.g. vulnerable young women [36].

### Feasibility

Feasibility refers to constraints at the personal and health system level that may impede the implementation of HIV treatment programmes. A recent study in South Africa assessed the human and financial resources requirement for different HIV treatment strategies [14] but overall there is little systematic guidance on how these constraints can be considered.

### Fundamental weaknesses of present approaches

The above overview shows a rapidly growing evidence-base on the efficiency, equity and feasibility of HIV treatment in South Africa. Yet, we argue there are two fundamental weaknesses to the current approaches that hamper policy makers in their ability to guide priority setting decisions.

Firstly, the HIV treatment programme in South Africa is not fully rational, with rational referring to “evidence-based allocation decisions in health taking into account all relevant decision-making criteria” [37]. The current programme is largely based on international guidelines and does not adequately account for aspects of efficiency, equity, and feasibility – these are not well documented, difficult to trade-off and therefore typically considered one at a time. For example, cost-effectiveness analyses consistently show that UTT is a highly efficient intervention but thereby ignore the severe health system capacity constraints of such a strategy, other than the budget [14]. As another example, the use of pre-exposure prophylaxis (PrEP) has shown to be effective and cost-effective to prevent HIV acquisition, but the community may prioritise to treat those people who are in greatest need of ART for their own health (even when this is less cost-effective). It is obvious that studies that fail to simultaneously consider efficiency, equity and feasibility concerns also fail to fully inform priority setting decisions [37]. Underlying reason is that studies are typically not multidisciplinary (they stem from either clinical medicine, epidemiology, health economics or ethics), nor interdisciplinary (little effort has been made to take into account community views) [37].

Second, HIV treatment priority setting is a highly political process but in the seemingly absence of fair participatory processes, stakeholders’ views are typically not incorporated. The legitimacy of decision-making in health refers to the use of “generally considered fair conditions for distributive decision-making in health” [38-40] corresponding to “the belief that authorities, institutions, and social arrangements are appropriate, proper, and just” [40]. Experience shows that there is often justifiable disagreement among stakeholders on which values to use in priority setting decisions [41,42]. Ethicists have realized there are no absolute truths on principles to guide priority setting decisions, and argue that decision-makers must rely instead on a fair process (i.e. procedural fairness) to establish fair decisions [43,44]. In contrast, the studies – as referred to above – typically rely on the assumption of ideal policy-makers, and that the mere provision of quantified evidence to policy makers leads to justified priority setting decisions.

**Figure 1:**
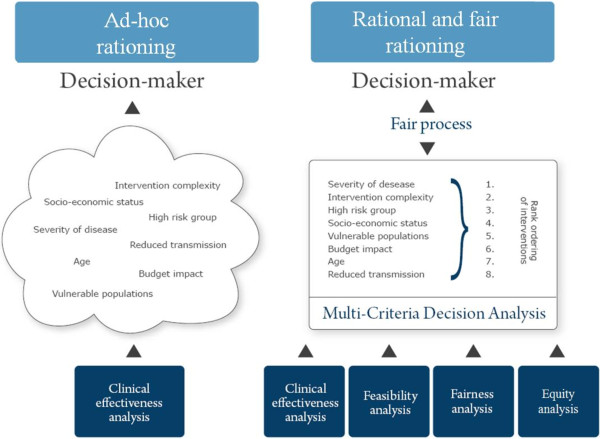
**Ad-hoc vs. rational and fair priority setting (based on Baltussen *et al.*)**[37].

The resulting picture is that of an ad-hoc priority setting process on HIV treatment (Figure 1, left panel).

### An alternative approach

Here we propose an alternative approach to provide a better evidence base and include a fairer policy process to improve HIV treatment priority setting in South Africa. It is based upon two innovative and increasingly important frameworks to health care priority setting: the ethical framework on *accountability for reasonableness (A4R)* that fosters fair priority setting, and *multi-criteria decision analysis* (*MCDA)* that fosters rational priority setting [37,45-56]. We believe that the integration of the two frameworks in a single approach holds large potential to improve fair and rational priority setting (Figure 1, right panel) [57]. Pilot studies show that decision makers support the principles of both frameworks [54,58].

### Accountability for reasonableness

A4R is generally considered as a leading conceptual framework on the ethics of health care priority setting. Based on justice theories of democratic deliberation, it aims to strengthen the fairness of priority setting decisions [43,44]. Central to the framework is the acceptance that people may justifiably disagree on what reasons to consider when priorities are made. In order to narrow the scope of controversy, A4R relies on “fair deliberative procedures that yield a range of acceptable answers” [43]. Therefore, A4R provides structure for decision-makers to establish priorities for their specific contexts, while taking into account limited resources and regulatory conditions. Its central notion of democratic learning presumes that a continuous participatory process will lead to better knowledge and consensus building on criteria for decisions, and thus also strengthening agreement on - or at least acceptance of - decisions. A4R does not replace any other guideline, planning or decision making process, but adds procedural principles to support their implementation. The A4R framework consists of four conditions.

•*Relevance.* Priority-setting decisions should be based on evidence, reasons and principles accepted by the stakeholders as relevant for meeting health needs fairly in their contexts. Closely linked to this condition is the inclusion of a broad range of stakeholders in the decision-making process. Having a wide range of stakeholders participating in the deliberation would help include the full range of relevant reasons and facilitate the implementation of the decisions made.

•*Publicity.* Decision makers should make the process of priority setting transparent including the reasons behind the decisions. This gives the general public an opportunity to understand the values of the choices involved and a possibility to assess whether the relevant procedures are being followed. Publicity is important because it facilitates comparison from case to case to ensure consistency, it invokes appeal and may improve trust.

•*Appeals/Revision.* The appeals condition is a mechanism that provides the public with an opportunity to dispute and challenge decisions. Thus, it also offers the decision makers an option to revise decisions in the light of further arguments. An appeals mechanism enriches decision-making process because initial agreement on reasons can be reassessed in light of new evidence.

•*Leadership/enforcement*. There must be public or voluntary regulation of the decision-making process to ensure that relevance, publicity and appeals mechanisms are enforced and that decisions are considered as fair. Proper enforcement of fairness in decision making will ensure that decisions are acceptable and can be supported by all concerned. Activities based on such decisions are also likely to be more effective and sustainable.

Many authors propose A4R as a guiding framework on the fairness of HIV treatment [33,35,41,42]. Yet, it has been criticised for being of limited practical use, i.e. for not detailing the 'Relevance conditions’, and how to include relevant evidence in the deliberation process [57,59]. Given the importance of A4R as conceptual framework in health care priority setting, there is an urgent need to put it into operation.

### Multi-criteria decision analysis

MCDA is theoretically grounded in multi-attribute utility theory [60] and sets programme priorities by referring to a comprehensive set of explicit criteria and guides decision makers in understanding the trade-offs between values that may be conflicting [37]. As such, it could be used in studies on priority setting of HIV treatment, to simultaneously consider efficiency, equity and feasibility concerns.

MCDA is routinely used in other disciplines like agriculture [61,62], as a response to the observed inability of people to effectively analyze multiple streams of dissimilar information, but knows relatively few applications in health. An example of the use of MCDA, in HIV treatment is a study by Cleary *et al.*, [41] in South Africa – who used mathematical programming techniques to trade-off equity and efficiency concerns. They estimated the health effects at different budget levels in the absence of any equity constraint (“health maximization”), and in the presence of two equity constraints: “equal treatment to all”, and “decent minimum”. The conclusion was that “health maximization” could achieve sizeable health gains but this would leave a quarter of those eligible for treatment without care. “Equal treatment” and a “decent minimum” would be more equitable but lead to less profound health gains. Other studies have used MCDA to set priorities in HIV/AIDS control in Thailand [51,53] and Indonesia [63,64].

A core component of any MCDA is the performance matrix which scores all programme options in terms of their performance on relevant criteria. Table 1 shows a hypothetical performance matrix for the evaluation of HIV treatment programmes. Each row describes a programme option (on *how* to deliver HIV treatment, *when* to initiate treatment, and *who* gets targeted for treatment) and each column describes the performance of the options against the criteria “feasibility”, “efficiency”, and “equity”. For example, mobile-clinic based treatment does not perform well on “efficiency” (as mobile clinics are relatively costly), but good on “equity” (as it is a way to reach remote areas and provide treatment to all). The matrix in Table 1 is highly simplified ― in reality, more criteria may be included which makes it adjustable to context. The matrix also quantifies the detailed performance on all criteria as well as trade-offs. For example, per programme option, the matrix may detail the number of life-years averted, among which population groups these occur, the expected costs, and required health system capacity―this allows a quantification of the trade-offs. In addition, in a real life application, the programme options in the performance matrix include coverage levels and can be combined.

**Table 1:** Hypothetical and simplified MCDA for HIV treatment in South Africa

**HIV treatment programme option**	**Feasibility**^**†**^	**Efficiency**^**†**^	**Equity**^**†**^	**Other**	**Total**^**‡**^	
	**Health system constraints**	**Acceptability**	**Costs per health gain**	**Fair distribution of health gains**	**…..**	
**How to deliver treatment**						
**Hospital-based treatment**	**••**	**••**	**••**	**••**		**••**
**Facility-based treatment**	**•••**	**•••**	**•••••**	**••••**		**••••**
**Mobile clinic-based treatment**	**•**	**••••**	**••**	**•••••**		**•••**
**Transport subsidies**	**••**	**••••**	**••**	**•••••**		**•••**
**When to initiate treatment**						
**Treatment CD4 < 200 cells/μl**	**•••••**	**•••**	**••**	**••••**		**•••**
**Treatment CD4 < 350 cells/μl**	**•••**	**•••**	**••••**	**•••**		**•••**
**Universal test and treat**	**•**	**••**	**•••••**	**•**		**••**
**Who gets targeted for treatment**						
**Discordant couples**^**‡‡**^	**•••**	**••**	**•••••**	**••**		**•••**
**Compliant patient groups**^**‡‡**^	**•••**	**••**	**•••••**	**•**		**•••**
**Pregnant women**^**‡‡**^	**•••**	**••••**	**•••**	**•••**		**••••**
**Productive adults**	**•••**	**•••**	**•••**	**•**		**••**
**First-come first-serve**	**•••**	**•**	**••**	**•**		**••**
**Weights**	**20**	**20**	**30**	**30**		

†The performance of interventions on efficiency, equity and feasibility is hypothetical and for iIIustrative purposes only. Criteria are example criteria only. The scoring ranges from • to ••••• respectively representing a very weak to very strong performance of an intervention on a certain criteria.

‡The total is calculated as the weighted scores on all criteria and rounded-off.

‡‡Irrespective of CD4 cell count.

There are several ways to interpret the performance matrix. In a qualitative inspection, any decision maker simply makes implicit judgments on the weights of the various criteria. Alternatively, in a quantitative inspection, any decision maker weighs the different criteria on the basis of their relative importance, and multiplies the scores by the weights to obtain weighted averages for all programmes. Programmes can subsequently be rank-ordered according to these weighted averages, somehow representing social welfare [53]. Table 1 shows hypothetical criteria weights at the bottom row, and weighted averages in the utter right column, to illustrate the latter; here facility-based treatment would be ranked first in the choice on 'how to deliver treatment’ [37].

### The contours of an alternative approach

The integration of the two frameworks in a single approach holds large potential to improve fair and rational priority setting [57]. While important frameworks on themselves, A4R should be informed by better evidence, and MCDA could be very useful in this regard if implemented in an accountable and transparent way.

**Figure 2:**
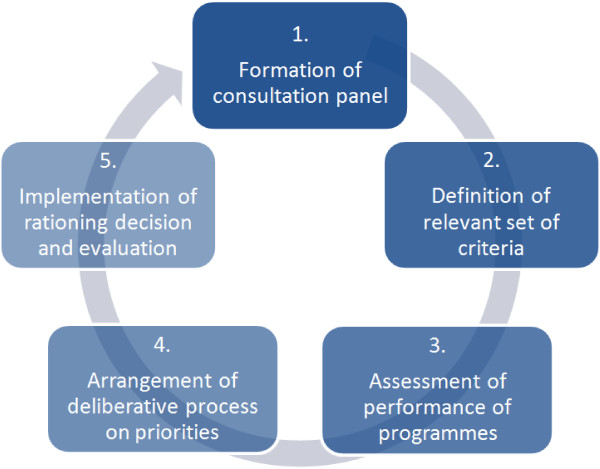
The health care priority setting process of HIV treatment programmes - an alternative approach.

The contours of an alternative approach, including five phases, are shown in Figure 2. A first phase involves the formation of a consultation panel consisting of all relevant stakeholders and this may include representatives from a broad range of parties, such as decision makers, community representatives, people living with HIV/AIDS, health professionals, etc., [48,53]. The formation of this panel can be a gradual process starting with the present decision-making body.

In a second phase, the panel identifies the decision-making criteria on the basis of local values. This involves a deliberative process in which panel members put forward relevant criteria (reasons) for priority setting and discuss reasons, principles, and evidence that each view as relevant to making fair decisions about priorities. These criteria are discussed, and ultimately approved or rejected, by other panel members. The aim is to reach acceptance on a set of criteria that are considered reasonable by all panel members. Sometimes this will be through consensus or through democratic voting but other times through hierarchical decision making. Even these cases can be compatible with A4R when all values and criteria have been deliberated in a fair way, the rationales for the decisions are made available and appeals can be made when the priority setting decision is implemented.

This paper proposes the use of criteria under the general headings of “efficiency”, “equity”, and “feasibility”, but these criteria are obviously not predetermined. Instead, these specific criteria should be defined by the consultation panel as an outcome of stakeholders’ discussions on which values they find most important in HIV treatment.

A third phase concerns the construction of the performance matrix, and this is the core component of any MCDA. In this step, all programme options are scored in terms of their performance on the selected criteria. In a fourth phase, the panel interprets the performance matrix. This may or may not involve the weighing of the relevant criteria. The A4R framework stipulates that this phase always includes a component of deliberation to discuss these weights, to identify any other criteria (that may have been ignored in the previous phases or that cannot be quantified), and to address the reasonability of the final ranking ordering. Phase five is the phase of evaluating the priority setting decision arrived at, and relates to the transparency, appeal and enforcement conditions of A4R (as described above).

Health care priority setting is a continuous process, where ethical dilemmas and programme priorities may regularly need to be updated in the light of changes of available programme options, of programme characteristics in terms of efficiency, equity and feasibility, or of stakeholders’ preferences. Health care priority setting is therefore represented here as a cyclical process. The cycle also reflects that health care priority setting is a (democratic) interactive learning process, in which the consultation panel constantly refines the participatory process of identifying, elaborating and deciding on the inclusion of further relevant stakeholders, criteria and evidence.

## Discussion

Balancing efficiency, equity and feasibility in priority setting of HIV treatment is a major challenge, and we have shown that present approaches fall short in adequately doing so in South Africa. We propose an alternative approach that integrates two existing frameworks, and believe this provides a better evidence base and outlines a fairer policy process to improve HIV treatment priority setting in South Africa.

This approach is innovative in a number of ways. Most importantly, the programme integrates separate disciplines of thought on health care priority setting in a single framework. The scientific literature of health care priority setting –whether it is in low-, middle- or high-income countries– typically does not go beyond the boundaries of traditional disciplines like medicine, epidemiology, health economics and ethics. Our suggested approach unites insights and methods from these disciplines, and merges disease modelling, cost-effectiveness analysis, equity analysis and procedural fairness in one single approach. The potential of merging approaches from different disciplines has been named before by Peacock *et al.*, who proposed a novel interdisciplinary framework combining MCDA, A4R, Participatory Action Research (PAR) and Programme Budgeting and Marginal Analysis (PMBA) [59]. However, to the best of our knowledge, this novel framework has not been explicitly put in practice. Yet, at the same time, PBMA is reported to routinely take care of many of the aspects raised above [65].

The approach can be implemented at different political levels in South Africa including national, province, district and community level. Here it provides support for management and a strategy for quality improvement in regard to health care priority setting, including a heretofore missing evidence-base for these decisions. By combining the MCDA and A4R, the approach incorporates many elements that bring a large capacity for considerations. Its implementation results in policies that are grounded on evidence-based research and that encourage involvement from all stakeholders. More importantly it may lead to a greater understanding and acceptability also from those directly affected by policy changes [57].

The use of our proposed approach in different decision-making contexts may lead to the inclusion of different stakeholders, identification of different criteria and ultimately to the selection of different interventions. While this may reflect the presence of different values in these different contexts, it may possibly also reflect differences in the rigor of implementation of the approach. The development of checklists on stakeholders and criteria (as suggested by Tromp and Baltussen) [66] to consider may reduce these latter differences. The use of a more standardized approach including a priori defined criteria (and possibly even criteria weights) would ignore differences in values in different contexts, and the importance of the deliberative process.

The integration of A4R and MCDA also poses a number of challenges. First, whereas A4R can be considered as a continuous democratic governance approach based on reasons that any stakeholders brings into play, MCDA requires a higher level of competence for its interpretation. This may run the risk of leaving out some stakeholders and limit the influence of others. Yet, first experiences on the use of MCDA did not identify this as a barrier in the process [48]. Second, the development of rigour evidence for health care priority setting, through MCDA, requires innovative research. Quantitative measures of equity and feasibility need to be developed, and measures of impact and efficiency need refinement. Also, mathematical models need to be developed that reflect the performance of treatment programmes in terms of efficiency and equity - these models could include measures of feasibility (as e.g. health workers availability) as health system capacity constraints. Yet, if the latter would be necessary in any health care priority setting process, MCDA runs the risk of needing a high level of expertise to provide credible evidence to the priority setting process. One way of addressing this is to allow, at least in the beginning of a process, more reliance on qualitative analysis within the consultation panel [67,68].

## Competing interests

The authors declare that they have no competing interests.

## Authors’ contributions

All authors contributed to the development of the conceptual framework, read and approved the final manuscript.
